# The Importance of an In-depth Study of Immunoglobulin Gene Rearrangements When Ascertaining the Clonal Relationship between Concomitant Chronic Lymphocytic Leukemia and Multiple Myeloma

**DOI:** 10.3389/fimmu.2016.00625

**Published:** 2016-12-27

**Authors:** Stéphanie Trudel, Hussein Ghamlouch, Julie Dremaux, Caroline Delette, Véronique Harrivel, Jean-Pierre Marolleau, Brigitte Gubler

**Affiliations:** ^1^Laboratoire d’Oncobiologie Moléculaire, Centre Hospitalier Universitaire Amiens Picardie, Amiens, France; ^2^EA 4666 Lymphocyte Normal – Pathologique et Cancers, Université de Picardie Jules Verne, Amiens, France; ^3^Service d’Hématologie Clinique, Centre Hospitalier Universitaire Amiens Picardie, Amiens, France; ^4^Laboratoire d’Hématologie, Centre Hospitalier Universitaire Amiens Picardie, Amiens, France

**Keywords:** concomitant hematological malignancies, clonal origin, immunoglobulin gene rearrangement, DNA copy number, cell sorting

## Abstract

Chronic lymphocytic leukemia (CLL) and multiple myeloma (MM) are hematological disorders that occur at different stages of B-cell development. It has been shown that CLL B-cells can differentiate into plasma cells *in vitro* and *in vivo*. CLL is the most frequent adult leukemia in the western world. It is a heterogeneous disease, characterized by clonal proliferation and the accumulation of mature CD5+ B lymphocytes (1). MM is a clonal plasma cell malignancy that accounts for more than 10% of all hematologic cancers (2). Although secondary cancers [particularly solid tumors (3–5)] can occur with CLL and MM, the concomitant occurrence of these two disorders in the same patient is rare [for a review of the few reported cases, see Ref. (6)]. The clonal relationship between these diseases has not always been clarified but is important in terms of understanding the pathogenesis and optimizing treatment. The clonal relationship between CLL and MM can be evaluated by (i) analyzing immunoglobulin (Ig) heavy chain and light chain (Ig kappa light chain and Ig lambda light chain) gene rearrangement, (ii) identifying and comparing somatic mutations, and (iii) studying chromosomic aberrations. Nevertheless, Ig rearrangements must always be interpreted in the light of specific phenomena such as allelic exclusion, B-cell receptor (BCR) revision (V_H_ and D_H_ gene replacement), BCR editing, and somatic mutations—events that were not considered in previous studies. These issues can be addressed by sequencing the rearranged Ig genes from sorted populations and interpreting the generated data. In the present study, we evaluated the putative clonal relationship between the two diseases by combining DNA copy number analysis with an assessment of Ig gene rearrangements [clonality assessment, V(D)J sequencing, and somatic hypermutation analysis] in highly enriched CD19+ CD5+ (CLL) and CD38+ CD138+ (MM) cell populations. Array comparative genomic hybridization data suggested a possible phylogenic progression from CLL to MM. Moreover, V(D)J sequencing indicated that both CLL and MM cells used the same V_H_ and J_H_ genes but different D_H_ genes. However, in-depth analysis and interpretation of Ig gene rearrangements ultimately suggested that the two diseases had distinct clonal origins.

## Case Presentation

In 2004, a 75-year-old woman was referred to our institution for monoclonal gammopathy. Laboratory screening revealed elevated serum levels of monoclonal IgG (20 g/l) and κ light chains (300 mg/l) in the absence of anemia and kidney failure. X-rays did not show any osteolytic lesions. A bone marrow biopsy revealed that 10% of the plasma cells were atypical. These results supported a diagnosis of Durie–Salmon stage I, International Staging System stage 1 IgG kappa multiple myeloma (MM). From 2004 to 2009, the patient did not present with an indication for treatment and so underwent regular clinical follow-up and laboratory tests. In October 2009, a complete blood count revealed an increase in the lymphocyte count (5.10^9^/l). Using flow cytometric immunophenotyping, the peripheral lymphocytes were positive for CD5, CD20, and CD23 (with expression of IgM and IgD lambda) and negative for CD38; this profile was consistent with a diagnosis of chronic lymphocytic leukemia (CLL; Matutes score: 5). A second bone marrow biopsy revealed major infiltration (59%) by atypical plasma cells and the presence of small, mature-looking lymphocytes with the same immunophenotype as peripheral lymphocytes. At this time, the patient did not present with lymphadenopathy, hepatosplenomegaly, or marked anemia and thrombopenia. We therefore diagnosed Binet stage A CLL in a patient with previously diagnosed MM. Given the lack of symptoms and the early disease stage, the patient did not require immediate treatment for CLL. Nevertheless, following an increase in the serum-free kappa light chain concentration (1000 mg/l) and the observation of rib fractures on chest X-rays, we decided to treat the patient with bortezomib–melphalan–prednisone. Meanwhile, the patient gave his written informed consent to performance of the molecular analyses and to publication of the present report.

## Materials and Methods

### Cell Isolation

Samples of peripheral blood and bone marrow were obtained (before treatment) from the patient. Peripheral blood mononuclear cells (PBMCs) and bone marrow mononuclear cells (BMMCs) were isolated using Ficoll-Hypaque™ density gradient centrifugation. CD19+ CD5+ CLL cells and CD38+ CD138+ MM cells were sorted using fluorescence-activated cell sorting (FACS) (Ariall, BD Biosciences). Genomic DNA was isolated from each sorted population using a DNeasy kit (Qiagen) and was employed in all subsequent molecular analyses.

### Clonality Assessment, V(D)J Sequencing, and Somatic Hypermutation Analysis

Genomic DNA samples from the cell-sorted populations underwent an immunoglobulin (Ig) heavy chain (IGH) and light chain [Ig kappa light chain (IGK) and Ig lambda light chain] gene rearrangement analysis using multiplex PCR and the standardized BIOMED-II Concerted Action protocol (7).

For the clonality assessment with fragment analysis, 1 µl of PCR product was mixed with 0.5 µl of a dye-labeled size standard [GeneScan™ 500 LIZ™ dye size standard (Applied Biosystems)] and 12 µl of deionized formamide (Hi-Di™ Formamide, Life Technologies), and denatured for 1 min at 95°C. Subsequently, fluorescent PCR products were separated by capillary electrophoresis on a 3500xL Dx Genetic Analyzer (Applied Biosystems) and sized using GeneMapper^®^ Software v4.1.

For the analysis of V, D, and J sequences, approximately 5 µl of purified PCR product were sequenced using BigDye^®^ Terminator v1.1 Cycle Sequencing Kit, according to the manufacturer’s instructions (Applied Biosystems). Electropherograms were analyzed with Sequencing Analysis v.3.7 software (Applied Biosystems), and sequence data were interpreted using the IMGT^®^ database^1^ (8) and the Basic Local Alignment Search Tool. The mutation frequency was calculated as the percentage of mutations per V_H_ sequence after the detection of mutations in both strands (sense and antisense).

### DNA Copy Number Analysis

Array comparative genomic hybridization experiments were performed in accordance with the manufacturer’s instructions (Agilent). Briefly, 500 ng of DNA were digested with *Rsa*/*Alu*, labeled with either Cy3-dCTP or Cy5-dCTP, and competitively hybridized with human reference genomic DNA (female) on 4 × 44 k comparative genomic hybridization (CGH) microarrays. The arrays were washed and scanned, and the images were extracted using Feature Extraction software. Data were analyzed with Cytogenomics software version 2.7.

## Results and Discussion

Plasma cells are terminally differentiated B cells. It has been shown that CLL B-cells can differentiate spontaneously into antibody-secreting plasma cells *in vivo* (9, 10) and *in vitro*, when exposed to various stimuli and can produce IgM, IgG, and IgA (11, 12). It is thus possible that the malignant plasma cells are differentiated or transformed CLL B-cells *(hypothesis 1, H1)*. The two malignancies may also arise independently from the same premalignant hematopoietic stem cell (HSC) *(hypothesis 2, H2)* or from an early-stage differentiated B-cell *(hypothesis 3, H3)*.

Our phenotyping data revealed that the CLL B-cells and malignant plasma cells expressed different Ig heavy and light chain isotypes (IgM and IgD lambda for the CLL B-cells, and IgG kappa for the malignant plasma cells), which suggested a different clonal origin. However, it has been shown that different Ig heavy and light chain phenotypes can arise from identical primary rearrangements and thus may correspond to a common clonal origin (13).

To establish whether MM cells are differentiated or transformed CLL B-cells (*H1*) or whether CLL and MM arose independently from the same premalignant HSC (*H2*) or from an early B-cell progenitor that had not begun to rearrange its Ig genes (*H3*), we decided to analyze the copy number variation (using array CGH) after cell sorting. Cytogenetic aberrations (such as deletions, loss of heterozygosity, duplications, or translocations) can drive carcinogenesis and cause clonal evolution, which is associated with disease progression and a poor clinical prognosis. Cytogenetic aberrations in HSCs occur in a number of hematological cancers, including CLL (14–16) and MM (17, 18). Indeed, the HSC’s genetic heritage is transmitted to the progeny; if this stem cell carries chromosomal aberrations that can be detected by array CGH, they should be present in the CLL and MM populations.

**Figure 1:**
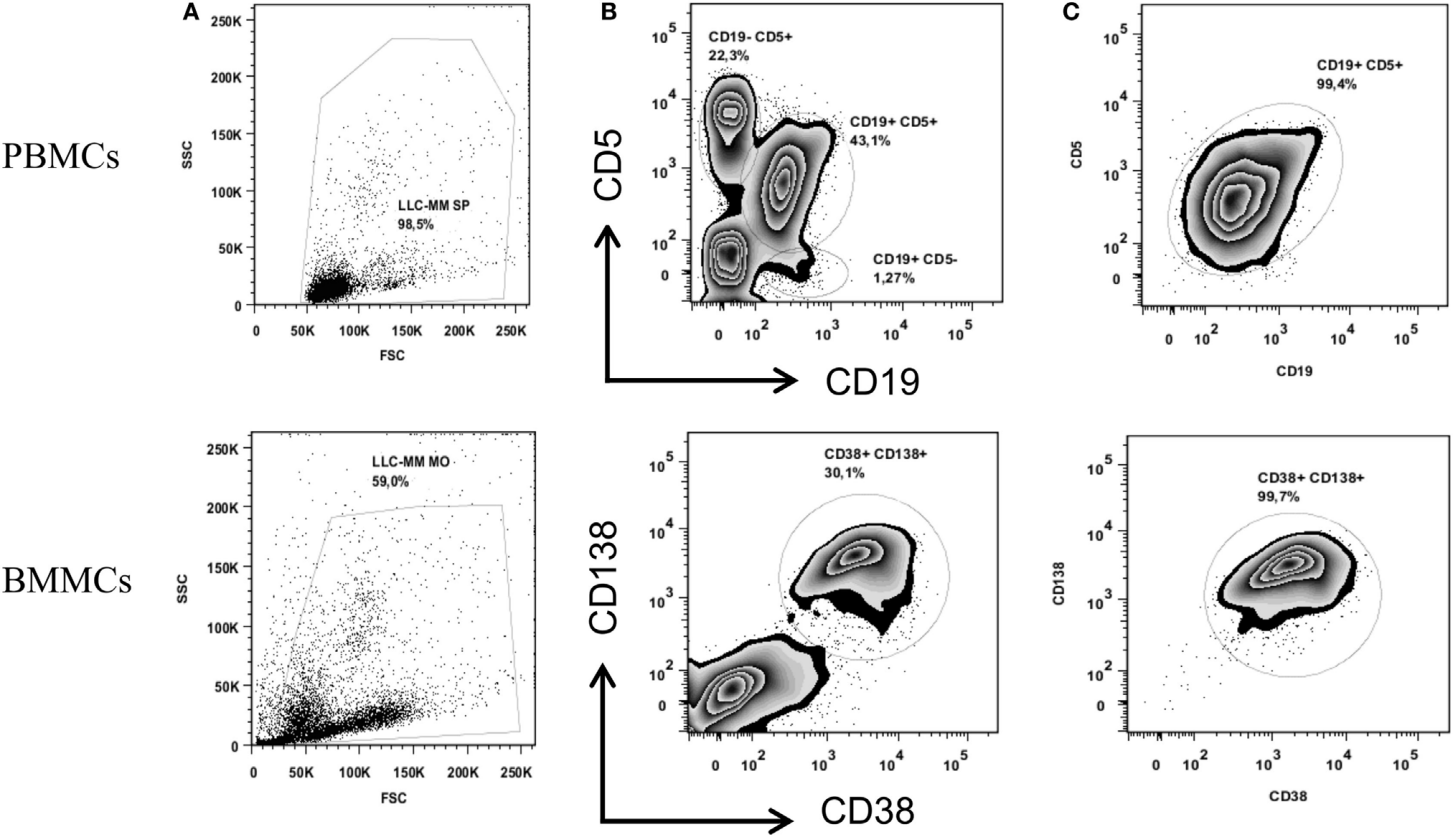
**Cell sorting of CD19+ CD5+ PBMCs (upper panel) and CD38+ CD138+ BMMCs (lower panel)**. Figures show the total cell population **(A)** and the percentage of the cell populations stained with anti-CD19 and anti-CD5 or anti-CD38 and anti-CD138 before sorting **(B)** and after sorting **(C)**.

**Figure 2:**
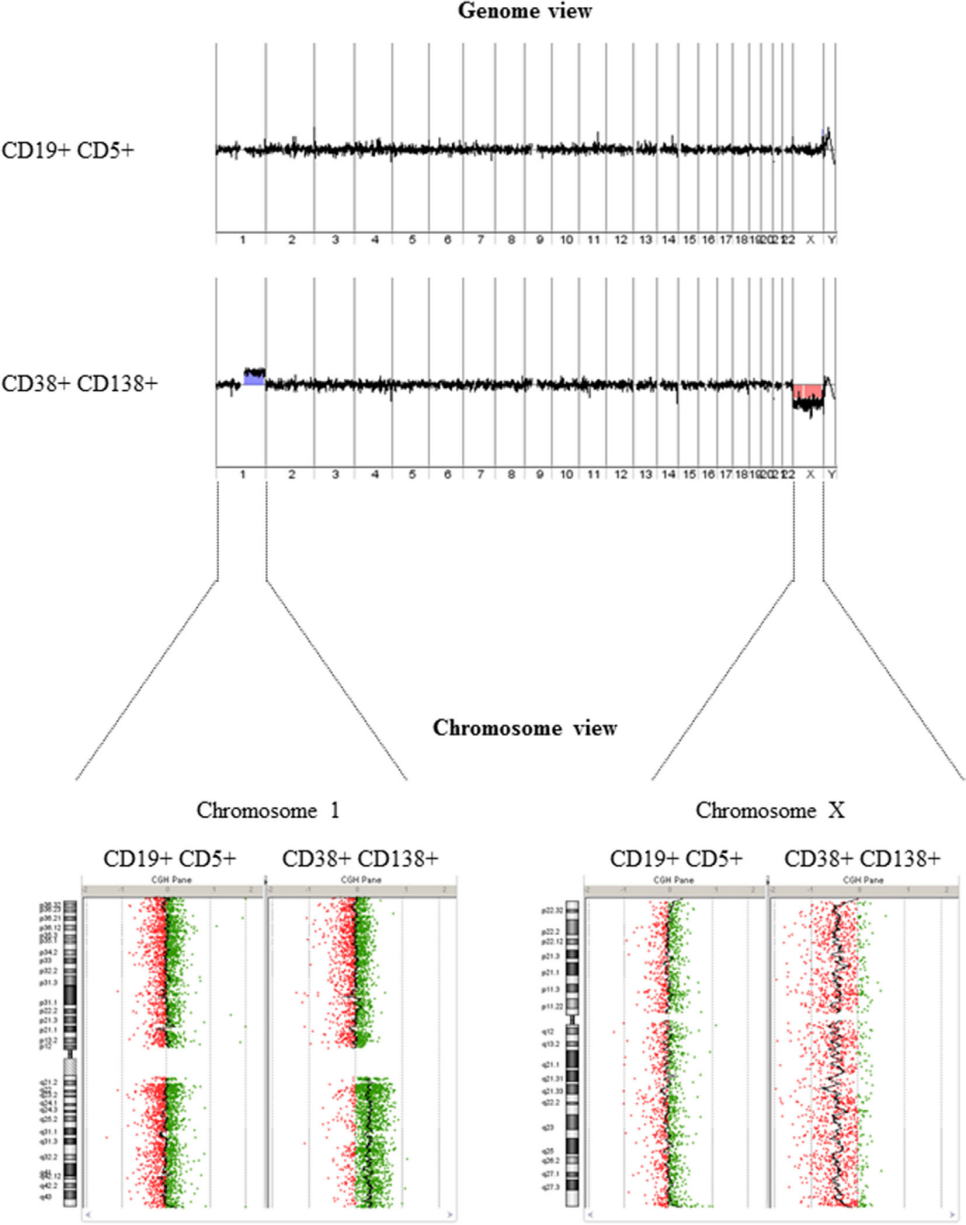
**DNA copy number analysis**. The genome view (upper panel) generated from array comparative genomic hybridization data did not show any detectable aberrations in DNA extracted from CD19+ CD5+ selected cells but revealed amplification/deletion events in DNA extracted from CD38+ CD138+ selected cells. A comparison of chromosome views (lower panel) shows a gain of the long arm of chromosome 1 (1q) (with a log ratio of +0.3) and a loss of chromosome X (with a log ratio of −0.3) in DNA extracted from CD38+ CD138+ selected cells.

After isolating PBMCs and BMMCs, we used FACS to obtain highly enriched CD19+ CD5+ (CLL) and CD38+ CD138+ (MM) cell populations (purity: 99.4 and 99.7%, respectively), as shown in Figure 1. A DNA copy number analysis revealed both amplifications (gain 1q21.1-q44, 104.1 Mb) and deletions (del X, 145.7 Mb) in CD38+ CD138+ sorted cells, whereas no aberrations were detected in CD19+ CD5+ sorted cells (Figure 2). The absence of common genomic aberrations in the two cell populations argues against the involvement of an early B-cell progenitor (*H2*). However, the detection of genetic aberrations in CD38+ CD138+ selected cells suggests that the malignant plasma cells were differentiated or transformed CLL B-cells (*H1*). This hypothesis can be evaluated by analyzing Ig heavy and light chain gene rearrangements.

During early B-cell differentiation in the bone marrow, IGH gene rearrangement precedes Ig light chain gene rearrangement. The D_H_ to J_H_ rearrangement occurs at the pro-B cell stage and produces an incomplete DJ_H_ rearrangement. This is followed by V_H_ to DJ_H_ rearrangement at the pre-B cell stage, which is thought to give rise to a complete, functional VDJ rearrangement. Following successful IGH recombination, the Ig light chain loci then rearrange themselves. After initial attempts occur at the Ig kappa locus (IGK); if a non-functional IgK or self-reactive rearrangement is achieved, the Ig lambda locus (IGL) then undergoes rearrangement. If VDJ_H_ and VJ_L_ are functional, the cell will develop into a mature naïve B-cell that co-express surface IgM/IgD; otherwise, the cell will undergo apoptosis or become anergic. When mature B-cells are activated by an antigen, they enter the germinal center, where somatic mutations and class-switch recombination occur. The B-cell goes through safety checkpoints at different stages of differentiation, and it may undergo additional rounds of rearrangement before (editing) or after (revision) it leaves the bone marrow (19, 20). As a result of the stepwise Ig gene rearrangement and accumulation of somatic V gene mutations, an analysis of the rearranged Ig genes in concomitant B-cell cancers provides information about the clonal relationship and the progenitor cell’s differentiation stage. Accordingly, two B-cells with the same IgH rearrangements but different IgL types may have a common clonal origin (13). In contrast, B-cells with different IgH rearrangements will always initially be considered to be biclonal.

**Figure 3:**
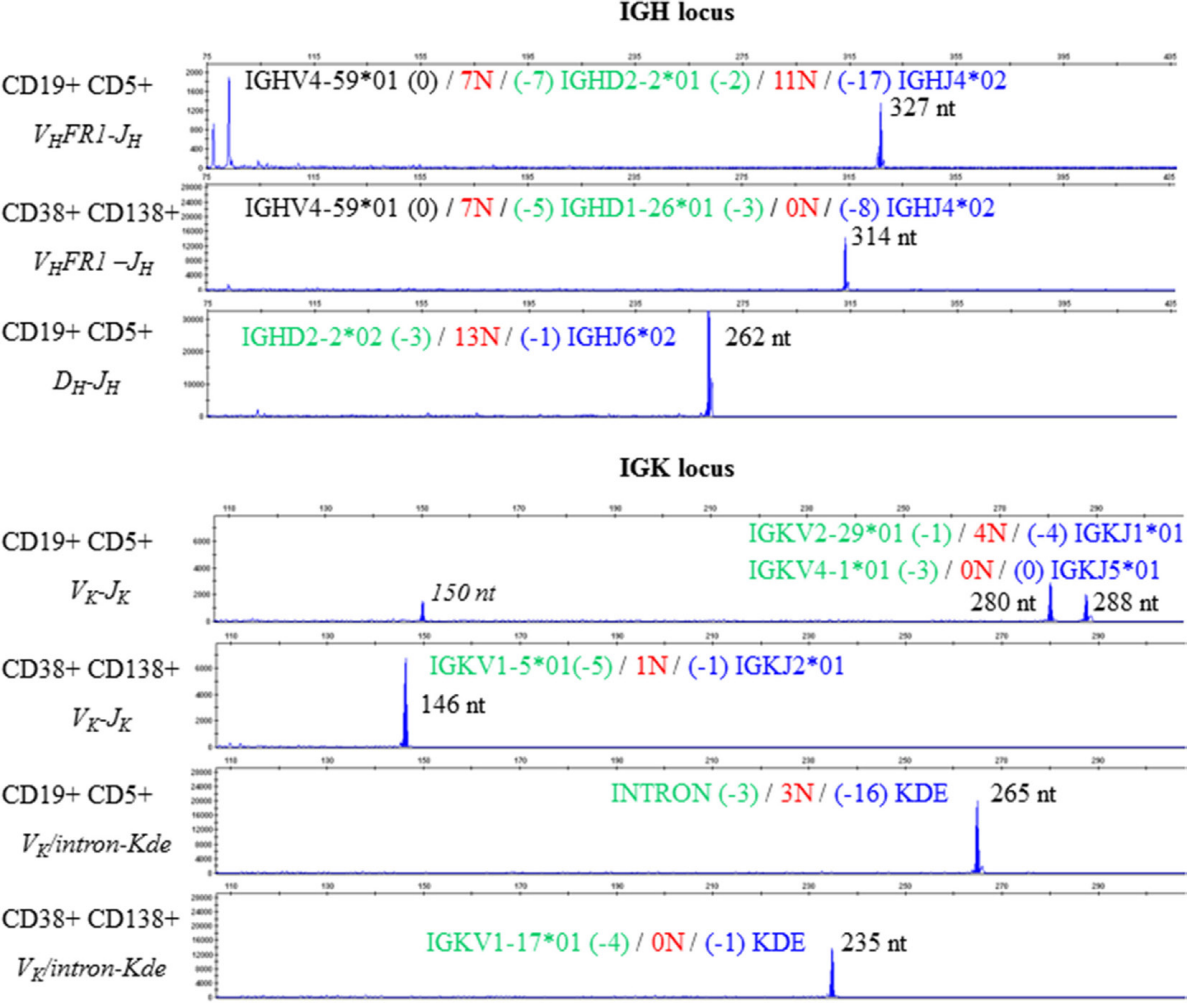
**Clonality assessment and V(D)J sequencing**. Electropherograms reveal different monoclonal peaks in the Ig heavy chain (upper panel) and Ig light chain kappa (lower panel) framework regions in CD19+ CD5+ PBMCs and in CD38+ CD138+ BMMCs. The sequence of each V(D)J rearrangement is indicated on the corresponding electropherogram.

We therefore studied IGH and light chain (IGK, IGL) gene rearrangements by analyzing genomic DNA from the cell-sorted populations. Assessment of the IGH locus identified monoclonal peaks in CD19+ CD5+ selected cells and in CD38+ CD138+ selected cells in multiplex V_H_FR1–J_H_, V_H_FR2–J_H_, and V_H_FR3–J_H_ PCRs (Figure 3, upper panel V_H_FR1–J_H_, data not shown for V_H_FR2–J_H_ and V_H_FR3–J_H_). A monoclonal peak was also detected using a multiplex D_H_–J_H_ PCR in the CD19+ CD5+ cell population. Analysis of the IGK locus using multiplex V_K_–J_K_ and V_K_/intron-Kde PCRs showed four monoclonal peaks in CD19+ CD5+ cells and two completely different peaks for CD38+ CD138+ (Figure 3, lower panel). Sequence analysis suggested that the PCR product with a peak size of 150 nt was a shorter amplification product of the peak at 288 nt. Multiplex PCR for the IGL locus displayed polyclonal Gaussian profiles for both populations (data not shown).

Sequencing of V_H_FR1–J_H_ PCR products from the CD19+ CD5+ and CD38+ CD138+ fractions revealed identical V_H_ (IGHV4-59*01) and J_H_ (IGHJ4*02) gene usage in both CLL and MM but different D_H_ gene usage (IGHD2-2*01 for CLL and IGHD1-26*01 for MM). In this context, one can imagine that a D_H_-replacement mechanism in a CLL B-cell gave rise to the V(D)J combination observed in the MM. D-replacement is a RAG-mediated, secondary recombination process that relies on the recognition of cryptic recombination signal sequences (RSSs) in the V_H_ element. It results in changes to the junction nucleotide sequences (21). We therefore analyzed the V_H_-3′region of each rearrangement and found that the cryptic RSSs and the surrounding region were conserved. These results strongly suggest (i) D_H_ replacement did not occur in this particular case and (ii) the two V(D)J rearrangements originated from independent clones (despite the same V_H_ and J_H_ gene usage). Moreover, the incomplete IGHD2-2/IGHJ6 rearrangement identified in CD19+ CD5+ cells could not have served as a basis for the complete IGHV4-59*01/IGHD1-26*01/IGHJ4*02 rearrangement observed in the MM; IGHJ6 is the most distal J_H_ gene in the IGH locus, and the IGHD2-2/IGHJ6 recombination process led to the deletion of all J_H_ genes^2^ (IGHJ1 to IGHJ5).

Chronic lymphocytic leukemia is believed to be driven by antigen–B-cell receptor (BCR) interactions (22). In this regard, studies have identified specific target such as bacterial antigen as well as auto-antigens that interact with CLL BCR. This notion is supported by the identification of closely homologous antigen binding sites between unrelated cases called stereotyped BCR (23). Stereotyped BCR uses a specific IGHV/IGHD/IGHJ genes rearrangement with nearly identical heavy and light chain complementarity-determining region 3 (CDR3) sequences and so identical antibody motifs. Up to one-third of patients with CLL have stereotyped BCR (23, 24). In this report, the usage of identical V_H_ and J_H_ genes was found in both CLL and MM. The sharing of motif that recognizes a same antigen may have driven the development of the two malignancies from independent B cell clones. However, the comparison of CDR3 length and the corresponding amino acid in CLL and MM, showed a higher CDR3 length in CLL and a different amino acid sequence, because of a different DH gene usage and the higher N diversity in DJ junction.

There are few literature reports of light chain gene rearrangement before heavy chain rearrangement (25). Therefore, we also considered the possible phylogeny of light chain rearrangements. Sequencing of both the rearrangements involving the Kde segment—one with the cryptic intron RSS and the removal of 16 nucleotides (in CLL) and the other with the IGKV1-17 gene and the deletion of one nucleotide (in MM)—confirmed the absence of common recombination events. Two V_K_J_K_ rearrangement configurations are possible in CLL, since recombination of IGKV4-1 segment occurs by inversion: the two V_K_J_K_ rearrangements can be located on the same haplotype or on different ones. If V_K_J_K_ rearrangements are located on the same haplotype, the IGKV4-1/IGKJ5 rearrangement necessarily preceded the IGKV2-29/IGKJ1 rearrangement; the latter would also have occurred by inversion in this new configuration. The IGKV1-5 and IGKJ2 genes are maintained on this haplotype and are thus available for subsequent rearrangement (*via* deletion) in MM. When considering possible phylogenies, the two CLL rearrangements would still be present in MM cells. However, IGKV1-5/IGKJ2 was the only rearrangement detected in CD38+ CD138+ cells—thus ruling out a common origin. However, if V_K_J_K_ rearrangements are located on different haplotypes (given that the IGKV2-29/IGKJ1 rearrangement deletes the IGKV1-5 segment), the IGKV1-5/JK2 rearrangement would be located on the haplotype bearing the IGKV4-1/IGKJ5 rearrangement. When considering possible phylogenies, the two CLL rearrangements would again still be present in MM cells, which is not what we observed.

Surprisingly, phenotyping data revealed a lambda monotype for CLL cells, whereas multiplex PCRs for the IGL locus displayed a polyclonal Gaussian profile in the two populations (data not shown). The high mutation frequency (>9%) observed for Ig gene rearrangements in the CLL B-cell population might explain why the clonal rearrangement was not amplified (i.e., no primer hybridization) and why the observed Gaussian profile could have come from polyclonal CD19+ CD5+ non-CLL B-cells. This discrepancy was partially resolved when sequencing studies revealed that IGKV2-29/IGKJ1 is an unproductive, rearranged IGK sequence (with a stop codon in CDR3 IGKV2-29). Even though the IGKV4-1/IGKJ5 rearrangement generated an open reading frame, the absence of kappa light chain production was certainly caused by the deletion of the kappa constant segment by the observed intron-Kde rearrangement.

In summary, our array CGH results in a highly purified cell population revealed that CLL cells did not have any detectable aberrations, whereas MM cells presented chromosome loss and gain. This prompted us to consider a possible phylogenic evolution from CLL to MM. In this particular context, only an in-depth analysis of Ig gene rearrangements could unambiguously determine the nature of the clonal relationship. A fragment analysis first revealed that the monoclonal components in CLL and MM differed in size. Nevertheless, when considering possible secondary recombination events (revision, editing, and replacement), this discrepancy did not rule out a primary common recombination event. This possibility was supported when IGH sequencing showed identical V_H_ and J_H_ gene usage in both CLL and MM cells. However, the observed D_H_ gene alignment and the lack of detectable secondary recombination events ruled out a transformation from CLL to MM (*H1*). Furthermore, it is unlikely that an early differentiated B-cell was a progenitor of both malignancies *(H3)* because common molecular stigmata would have been seen. Given the absence of a clonal relationship between CLL and MM, the most likely hypothesis (*H2*) involves the existence of a premalignant HSC (although our present data cannot prove this and cannot exclude the possibility that the two malignancies derived from independent HSC).

In patients with concomitant hematological diseases, the presence or absence of a clonal relationship can be formally addressed by combining an in-depth analysis of Ig gene rearrangements with a DNA copy number analysis in a highly purified, cell-sorted population.

## Ethics Statement

Ethical approval is not appropriate. The patient gave his written informed consent to performance of the molecular analysis and to publication of this case report.

## Author Contributions

ST and HG acquired, analyzed and interpreted the data, conceived and designed the case report, and drafted the manuscript. JD carried out the molecular analysis and helped to draft the manuscript. CD helped to draft the manuscript. VH interpreted phenotyping data. J-PM was in charge of the clinical follow-up, provided and interpreted the clinical data, conceived and designed the case report, and revised the manuscript. BG helped to analyze and interpret data, conceived and designed the case report, and corrected and revised the manuscript. All the authors read and approved the final manuscript.

## Conflict of Interest Statement

The authors declare that the research was conducted in the absence of any commercial or financial relationships that could be construed as a potential conflict of interest.

## Footnotes

^1^http://www.imgt.org.

^2^http://www.imgt.org/IMGTrepertoire/LocusGenes/#B.

## Abbreviations

BCR, B-cell receptor; BMMC, bone marrow mononuclear cell; CDR3, complementarity-determining region 3; CGH, comparative genomic hybridization; CLL, chronic lymphocytic leukemia; FACS, fluorescence-activated cell sorting; HSC, hematopoietic stem cell; IGH, immunoglobulin heavy chain; IGK, immunoglobulin kappa light chain; IGL, Ig lambda locus; MM, multiple myeloma; PBMC, peripheral blood mononuclear cell; RSS, recombination signal sequence.
